# The Association Between Metabolic Syndrome, Hyperfiltration, and Long-Term GFR Decline in the General Population

**DOI:** 10.1016/j.ekir.2023.06.022

**Published:** 2023-07-01

**Authors:** Erikka W. Bystad, Vidar T.N. Stefansson, Bjørn O. Eriksen, Toralf Melsom

**Affiliations:** 1 Metabolic and Renal Research Group, UiT-The Arctic University of Norway, Tromsø, Norway; 2 Section of Nephrology, Clinic of Internal Medicine, University Hospital of North Norway, Tromsø, Norway

**Keywords:** chronic kidney disease, general population, GFR decline, glomerular hyperfiltration, metabolic syndrome, renal hyperfiltration

## Abstract

**Introduction:**

One-quarter of adults worldwide meet the criteria of metabolic syndrome (MetS). MetS increases the risk of diabetes, chronic kidney disease (CKD), and cardiovascular disease. However, the association between MetS, hyperfiltration, and long-term glomerular filtration rate (GFR) decline in the general population is unknown.

**Methods:**

In the Renal Iohexol Clearance Survey (RENIS), we investigated 1551 people aged 50 to 63 years; representative of the general population without diabetes, cardiovascular disease, or kidney disease. The GFR was measured using iohexol clearance at baseline and twice during 11 years of follow-up. Hyperfiltration at baseline was defined as an absolute GFR (ml/min) above the 90th percentile adjusted for sex, age, and height, because these variables correlate with nephron number. MetS was defined as increased waist circumference and 2 risk factors among hypertension, hyperglycemia, elevated triglycerides, and low high density lipoprotein (HDL)-cholesterol levels. The GFR decline rate was calculated using linear mixed models.

**Results:**

MetS was associated with hyperfiltration at baseline (odds ratio [OR] 2.4; 95% CI: 1.7–3.5, *P* < 0.001) and a steeper GFR decline rate during follow-up (−0.30 [−0.43 to −0.16] ml/min per 1.73 m^2^/yr). Compared to those without MetS, GFR decline was −0.83 (95% CI: −1.13 to −0.53) ml/min per 1.73 m^2^/yr in those with MetS and baseline hyperfiltration and −0.15 (−0.30 to 0.00) in those MetS without hyperfiltration, *P* = 0.2 for interaction.

**Conclusions:**

In the nondiabetic general population, those with MetS had an increased OR of hyperfiltration and steeper long-term GFR decline. Randomized controlled trials are needed to explore whether treatment of hyperfiltration can prevent loss of GFR in persons with MetS.

## Graphical abstract

**Figure:**
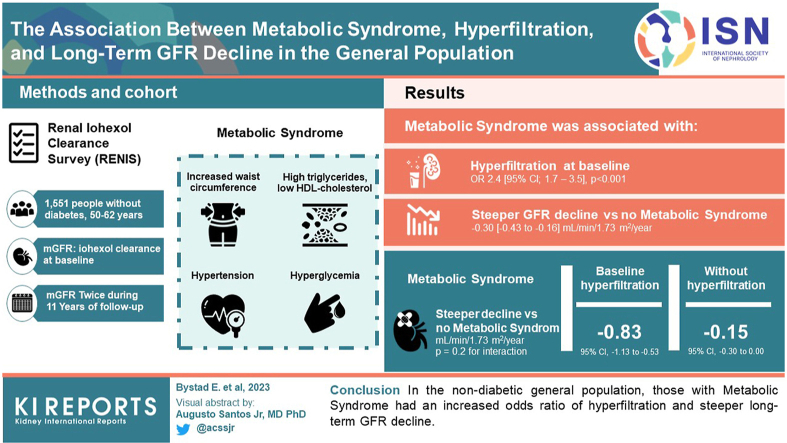

CKD affects more than 10% of the population globally and has become one of the leading causes of mortality worldwide.^1^ Hypertension, hyperglycemia, and obesity are important risk factors for CKD.^2^ These metabolic abnormalities are often clustered together in MetS.^3^ MetS is a risk factor for diabetes, cardiovascular disease, and CKD, therefore representing a window of opportunity for early preventive measures.^4^^,^^5^ Approximately one-quarter of the world population, and one-third of US adults meet the criteria of MetS, making it a global epidemic.^4^

Abnormally elevated single-nephron GFR or glomerular hyperfiltration has been proposed as a common pathway leading to glomerulosclerosis and the progression of CKD.^6^ At the whole-kidney level, renal hyperfiltration often precedes kidney disease in diabetes and may have a similar role in nondiabetic CKD. Renal hyperfiltration has been linked to components of MetS, such as prediabetes, hypertension, and waist circumference in some,^7^, ^8^, ^9^ but not in all studies.^10^, ^11^, ^12^ Similarly, the results from a few studies that investigated the association of MetS with renal hyperfiltration were divergent.^10^, ^11^, ^12^

Notably, previous general population studies were limited by the use of estimated GFR (eGFR) or creatinine clearance.^11^, ^12^, ^13^, ^14^, ^15^ The use of eGFR is problematic in studies of renal hyperfiltration because eGFR lacks precision in the high GFR range and is biased by non-GFR related metabolic risk factors, such as muscle mass, obesity, inflammation, and insulin resistance.^16^, ^17^, ^18^ In addition, eGFR is commonly normalized to the body surface area (BSA), which masks hyperfiltration in obese persons.^19^^,^^20^

Treatment with sodium-glucose cotransporter-2 inhibitors prevents GFR decline in persons with diabetes and CKD, likely partly because of attenuation of hyperfiltration.^21^^,^^22^ In a previous study, we found that, among the general nondiabetic population, those with a higher baseline measured GFR (mGFR) had a subsequent steeper GFR decline, similar to what was found in Pima Indians with diabetes, using the same analyses.^23^ In the current study, we hypothesize that MetS is associated with baseline hyperfiltration and that those with MetS and hyperfiltration define a subgroup with a high risk of accelerated long-term GFR decline. Our aim was to investigate the relationship between MetS, renal hyperfiltration, and long-term GFR decline using measurements of GFR in a representative sample of middle-aged Europeans without diabetes.

## Methods

### Study Population

**Figure 1:**
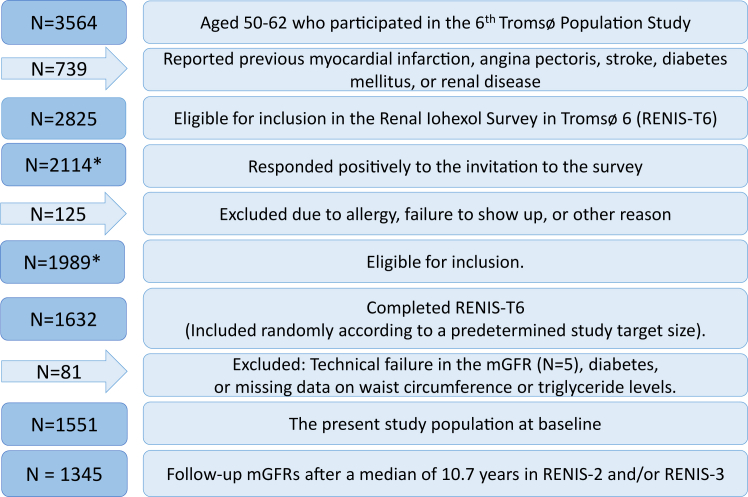
Inclusion of subjects in the RENIS. ∗Miscount in previous publications (previous numbers are 2107 and 1982, the correct numbers are 2114 and 1989, respectively). RENIS, Renal Iohexol Clearance Survey. RENIS, Renal Iohexol Clearance Survey.

RENIS-T6 was a substudy of the sixth population-based Tromsø study (Tromsø 6) conducted from November 2007 to June 2009. Forty percent of inhabitants aged 50 to 59 years and all inhabitants aged 60 to 62 years in Tromsø were invited to the Tromsø 6 study. In these age groups, 3564 (65%) completed the main part of Tromsø 6. Of these, we excluded 739 who reported a previous myocardial infarction, angina pectoris, stroke, diabetes mellitus, or renal disease. The remaining 2825 persons were invited to participate in RENIS-T6 and 2114 (75%) responded positively. Forty-eight persons withdrew after their first consent and 77 persons were excluded because of possible allergic reactions to contrast media. Out of 1989 eligible persons, we included 1632 individuals to RENIS-T6 in random order according to a predetermined target size. Subjects with a technical failure in the GFR measurement (*N* = 5), undiagnosed diabetes mellitus or lacking information about waist circumference or triglyceride levels were excluded, leaving 1551 participants eligible for our study (Figure 1). A total of 1345 (87%) had at least 1 mGFR at follow-up as part of the RENIS-2 and RENIS-3 studies after a median follow-up of 10.7 (6.3–11.3) years.^24^ The participants in the RENIS cohort were representative of all persons eligible for inclusion.^25^

The RENIS study was approved by the local ethics committees and performed in accordance with the guidelines of the Declaration of Helsinki. All subjects provided written informed consent.

### Measurements

The RENIS was conducted at the Clinical Research Unit of the University Hospital of Northern Norway. The participants fasted overnight. Participants with intercurrent disease were rescheduled to a later appointment.

Waist circumference and height were measured at baseline as a part of the Tromsø 6 study. Waist circumference was measured horizontally over the umbilicus after exhalation and height was measured to the nearest centimeter with a wall-mounted measuring tape. Body weight was measured in the RENIS-T6 study using the same digital scale for all subjects. Further description of body measurements is described by Stefansson *et al.*^26^

Blood pressure was measured 3 times using an automated device (model UA799; A&D, Tokyo, Japan), and the last 2 readings were averaged. Serum samples for triglycerides, cholesterol, and glucose levels were measured on a Modular P800 (Roche Diagnostics, Mannheim, Germany).

All participants completed a health questionnaire including tobacco use and all currently used medications. Smoking was dichotomized as current daily smoking (yes/no).

### The GFR

The GFR was measured using single-sample plasma clearance of iohexol at baseline and follow-up as described in detail previously.^24^^,^^25^ This method has been validated against gold standard methods and was recently found to show substantial agreement with the multiple-sample method.^27^^,^^28^

We measured serum creatinine and cystatin C as previously reported.^25^ eGFR was calculated using the 2009 and 2012 Chronic Kidney Disease Epidemiology Collaboration (CKD-EPI) equations for creatinine (eGFRcrea), cystatin C (eGFRcys), and both combined (eGFRcreacys).^29^

### Definition of Hyperfiltration

There is no consensus on defining renal hyperfiltration.^30^ Different GFR cut-offs, such as an absolute GFR >140 ml/min and age-specifiic and sex-specific percentiles, for example, above the 95 percentile, have been used in different studies.^30^ Because the RENIS included a moderate number of persons, we defined hyperfiltration as an absolute mGFR (ml/min) above the 90th percentile after adjusting for gender, age, and height.^7^^,^^8^ We adjusted for gender, age, and height to obtain a better proxy for single nephron hyperfiltration because these variables correlate with nephron number.^31^, ^32^As described in previous RENIS publications, we selected all subjects above the 90th percentile in the distribution of residuals from a multiple linear regression analysis in which we used the logarithm of absolute GFR as the dependent variable and sex, the logarithm of age, and height as independent variables.^7^^,^^8^ In secondary analyses, we assessed the association between the MetS and BSA-normalized mGFR, and between MetS and hyperfiltration defined by GFR (ml/min per 1.73 m^2^) above the 90th percentile after adjusting for the logarithm of age and sex, but not height, using the method as mentioned above.

For comparison, we also defined hyperfiltration using eGFR based on creatinine, cystatin C, and both combined, with and without BSA adjustment similarly as described above. To assess absolute eGFR (ml/min) we multiplied the eGFR (ml/min per 1.73 m^2^) with each participant’s BSA (= 0.07184 × weightˆ0.425 × heightˆ0.725).^33^

### Definition of MetS

MetS was defined as a dichotomous variable (yes/no) based on the International Diabetes Federation definition from 2006 shown in Table 1.^3^ In secondary analyses, we used the less strict National Institutes of Health cut-off values for waist-circumference (≥102 cm in men and ≥88 cm in women).^34^ We also repeated the analyses using a consensus definition incorporating the International Diabetes Federation and the American Heart Association/National Heart, Lung, and Blood Institute definition, where meeting any 3 of 5 criteria would qualify a person for the MetS (Supplementary Table S1).^35^ We excluded subjects with diabetes because of the known association with hyperfiltration.

**Table 1:** The International Diabetes Federation definition of metabolic syndrome

Waist circumference ≥ 94 cm in men and ≥80 cm in women along with the presence of 2 or more of the following:
• Fasting plasma glucose ≥5.6 mmol/l
• Systolic blood pressure ≥130 mmHg, diastolic blood pressure ≥85 mm- Hg or use of antihypertensive medication, or a combination of these
• Triglycerides ≥1.7 mmol/l or use of triglyceride-altering drugs
• HDL-cholesterol <1.03 mmol/l in men or <1.29 in women or use of HDL-altering drugs
The National Institutes of Health cut-off values for increased waist circumference (secondary analysis):
• ≥102 cm in men and ≥88 cm in women

HDL, high density lipoprotein.

### Statistical Methods

Pearsons X^2^ test, Welch’s t-test, and the Mann-Whitney U test were used to test the differences between the groups with and without MetS and sex differences in the prevalence of different MetS criteria.

The cross-sectional associations among MetS, its individual components, and hyperfiltration were investigated with absolute GFR (ml/min) as a continuous dependent variable using linear regression and as a dichotomized dependent variable (hyperfiltration: yes/no) using logistic regression. We adjusted for age, sex, and height because these factors are correlated with nephron number and may confound the association between MetS and glomerular hyperfiltration at a single nephron level.^31^ We also adjusted for smoking because it has been associated with both MetS and hyperfiltration.^36^ We did not adjust for body weight in the primary analyses because doing so will mask hyperfiltration in obese persons.^19^^,^^20^

The association of MetS at baseline with GFR decline rates was examined using separate linear mixed regression models with random intercept and slope and an unstructured covariance matrix. MetS was included as a dichotomous (yes/no) and categorical independent variable (No MetS, MetS without hyperfiltration, and MetS with hyperfiltration). The interactions between independent variables and time were taken to represent the independent variables’ associations with the GFR change rate.^37^ All 1551 participants were included in the analysis regardless of the number of follow-up GFRs because the linear mixed regression accounts for missing values.^38^ Effect modification of sex and hyperfiltration on the association between MetS and GFR decline rate was tested using a 3-way interaction term between MetS, time, and sex or hyperfiltration, respectively.

We used Stata software version 15 (Stata Corp., College Station, TX) for the statistical analysis. Statistical significance was set at *P* < 0.05. See Supplementary Material for the STROBE statement.

## Results

### Study Population Characteristics

The characteristics of the study population are presented in Table 2. Out 1551 participants, 479 (31%) met the criteria for MetS (299 [39.5%] men and 180 women [22.7%], *P* < 0.001 for sex difference). More women met the waist criteria of MetS (22.4% vs. 12.2%, *P* < 0.001) and more men met the glucose criteria (39.9% vs. 18.4%, *P* < 0.001), triglycerides criteria (22.4% vs. 12.2%, *P* < 0.001), and blood pressure criteria (68.8% vs. 49.9%, *P* < 0.001) for MetS. Subjects with MetS were, on average, older, heavier, had a higher body mass index, and had higher absolute GFR and BSA-normalized mGFR (all *P* < 0.05) (Table 2). In contrast to the mGFR, BSA-normalized eGFR (ml/min) using creatinine, cystatin C, and both were lower in the group with MetS.

**Table 2:** Characteristics of the study population at baseline

Variable	No Metabolic Syndrome	Metabolic Syndrome	*P* Value
Subjects	1072 (69.1%)	479 (30.9%)	
Male gender	458 (42.7%)	299 (62.4%)	<0.001
Age (yrs)	58.4 (51.5–63.1)	59.3 (51.6–63.2)	0.01
mGFR (ml/min per 1.73 m^2^)	93.1±14.3	95.2 ± 14.5	0.01
mGFR (ml/min)	100.3 ± 19.0	111.2 ± 19.9	<0.001
eGFRcrea (ml/min per 1.73 m^2^)	95.1 ± 9.3	94.1 ± 10.1	0.05
eGFRcrea (ml/min)	102.3 ± 13.9	109.9 ± 15.3	<0.001
eGFRcys (ml/min per 1.73 m^2^)	106.0 ± 12.0	103.6 ± 13.0	<0.001
eGFRcys (ml/min)	114 ± 18.0	121.2 ± 19.3	<0.001
eGFRcreacys (ml/min per 1.73 m^2^)	103.6 ± 11.2	101.3 ± 11.8	<0.001
eGFRcreacys (ml/min)	111.5 ± 16.3	118.4 ± 17.6	<0.001
Weight (kg)	75.47 ± 12.9	88.6 ± 12.9	<0.001
Height (cm)	169.7 ± 8.7	172.7 ± 8.5	<0.001
Body mass index (kg/m^2^)	26.1 ± 3.6	29.7 ± 3.6	<0.001
Current smoking	225 (21.0%)	85 (17.7%)	0.1
Office diastolic BP (mm Hg)	81.4 ± 9.4	87.7 ± 8.9	<0.001
Office systolic BP (mm Hg)	126.0 ± 16.8	137.1 ± 16.7	<0.001
ACE-inhibtor use, yes	13 (1.2%)	15 (3.1%)	0.01
Angiotensin receptor blocker use, yes	52 (4.9%)	76 (15.9%)	<0.001
Calsium-blocker use	39 (3.6%)	39 (8.1%)	<0.001
Beta-blocker use	32 (3.0%)	35 (7.3%)	<0.001
Diuretica use	68 (6.3%)	68 (14.2%)	<0.001
Waist circumference (cm)	91.5 ± 10.8	102.7 ± 9.5	<0.001
HDL-Cholesterol (mmol/l)	1.6 ± 0.4	1.3 ± 0.3	<0.001
Triglycerides (mmol/l)	0.9 (0.5–1.6)	1.6 (0.7–3.1)	<0.001
Fasting glucose (mmol/l)	5.2 ± 0.4	5.7 ± 0.4	<0.001
Fulfilled metabolic syndrome criterion			
Blood pressure criterion	499 (46.5%)	418 (87.3%)	<0.001
Triglyceride criterion	42 (3.9%)	225 (47.0%)	<0.001
HDL criterion	61 (5.7%)	178 (37.2%)	<0.001
Glucose criterion	125 (11.7%)	323 (67.4%)	<0.001
Waist circ. criterion	753 (70.2%)	479 (100%)	<0.001

ACE, angiotensin converting enzyme; BP, blood pressure; eGFR, estimated glomerular filtration rate; eGFRcrea/cys/creacys, estimated GFR based on creatinine, cystatine, and both combined; HDL, high density lipoprotein; LDL, low density lipoprotein; mGFR, measured glomerular filtration rate.

Data is presented as the mean (SD) for continuous variables with symmetric distributions, median (IQ range) for continuous variables with skewed distributions, and *n* (%) for categorical/dichotomous variables. No missing data.

### Association Between MetS and GFR

**Figure 2:**
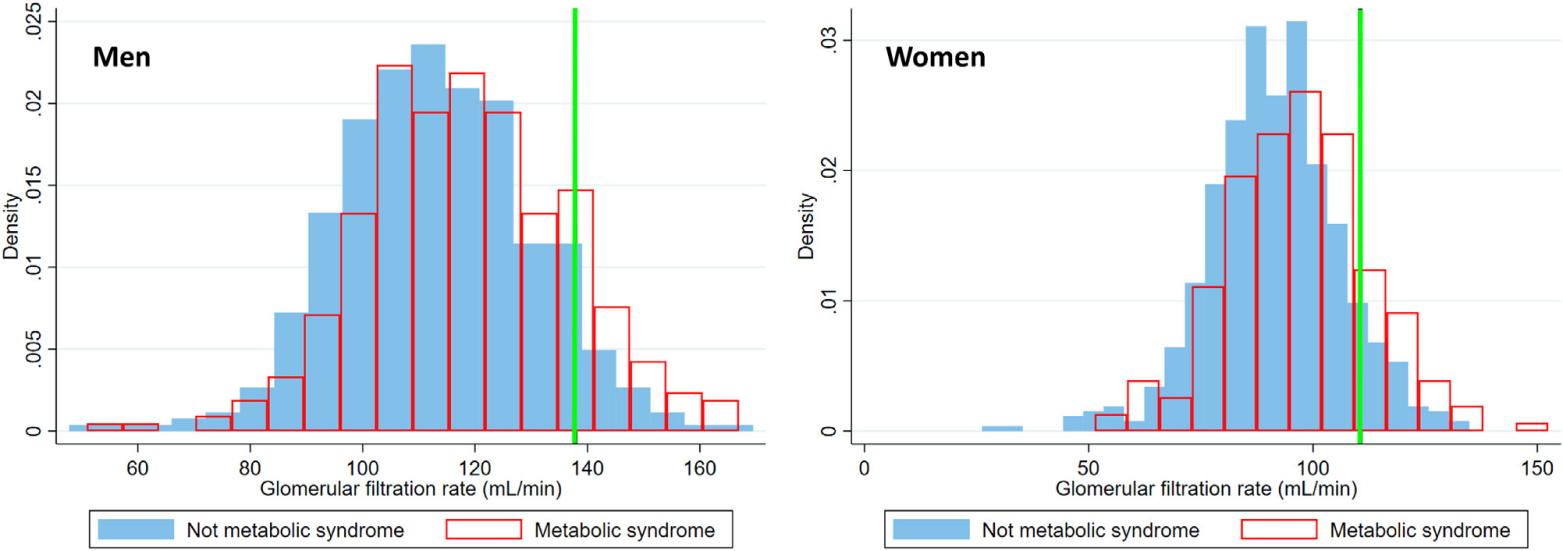
The distribution of GFR for those with and without MetS (red and blue) is shown separately for women and men. The GFR cut-off points for hyperfiltration (vertical green line) for a person with average age and height (111 ml/min in women and 138 ml/min in men). GFR, glomerular filtration rate; MetS, metabolic syndrome.

**Figure 3:**
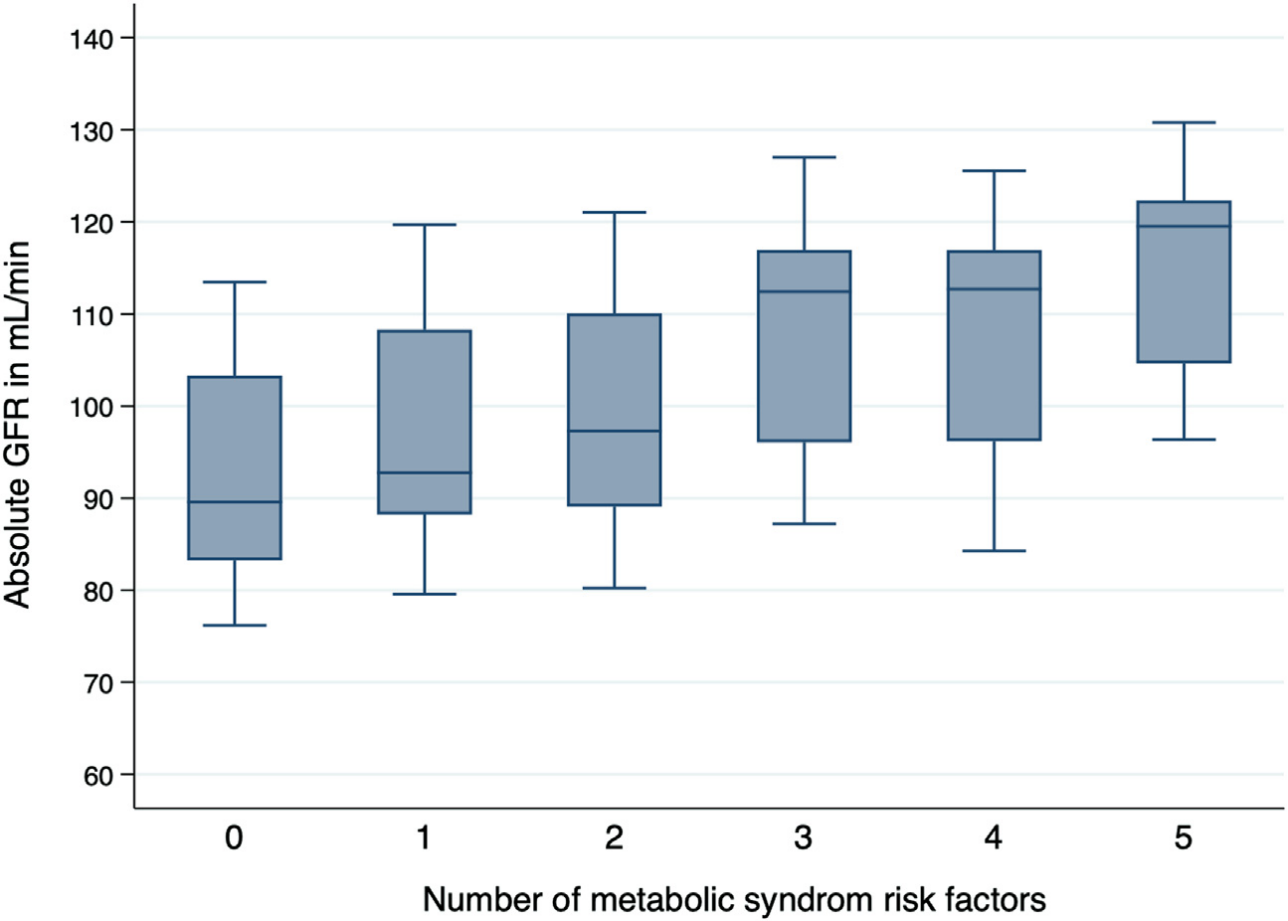
Absolute GFR by number of metabolic risk factors calculated using ANOVA, adjusted for age, sex and height. *P* value <0.001. GFR, glomerular filtration rate. ANOVA, analysis of variance; GFR, glomerular filtration rate

The distribution of absolute GFR (ml/min) for those with and without MetS is shown separately for women and men in Figure 2. Study participants with MetS had 6.7 (95% CI: 5.0–8.4) ml/min higher mean absolute mGFR than those without MetS in multiple linear regression adjusted for age, sex, height, and current smoking (Supplementary Table S2). Four of 5 components of MetS (all except hypertension) were individually associated with higher absolute mGFR. When the study population was divided into 6 subgroups according to the number of MetS criteria they met (0–5), mGFR (adjusted for age, sex, and height) increased with the number of criteria met (Figure 3).

### Association Between MetS and Hyperfiltration

By definition, 155 subjects (10%) were classified with hyperfiltration, of whom 53% were women. The GFR cut-off points for hyperfiltration for a person with average age and height were 111 ml/min in women and 138 ml/min for men (Figure 2).

Study participants with MetS had an increased OR of hyperfiltration (adjusted OR 2.5, 95% CI: 1.8–3.6) (Table 3). Hypertension was the only MetS criterion not associated with hyperfiltration (Table 3). Among the individual criteria, the waist circumference criterion was associated with the highest odds of hyperfiltration, followed by the triglycerides and glucose criteria. The association between hyperfiltration and the number of MetS criteria met is shown in Supplementary Table S3. The adjusted OR of hyperfiltration for those who met 1, 2, and 3 out of the waist criterion, glucose criterion, and triglyceride criterion compared to none were 2.8 (1.24–6.12), 6.0 (2.7–13.4), and 7.2 (2.9–18.0), respectively (Supplementary Table S4). The association of hyperfiltration with MetS (Table 3) remained almost the same after additional adjustment for high-sensitivity C-reactive protein (OR 2.52, 95% CI: 1.78–3.58). Further adjustment by angiotensin converting enzyme inhibitor use or angiotensin receptor blocker use in model 3 did not affect the association between hypertension and hyperfiltration (OR 1.1, *P* = 0.6).

**Table 3:** Associations of hyperfiltration with MetS and its components

Components of MetS	Model 1			Model 2			Model 3		
	OR	CI	*P* value	OR	CI	*P* value	OR	CI	*P* value
Metabolic syndrome, yes	2.44	(1.75–3.41)	<0.001	2.55	(1.81–3.60)	<0.001	2.54	(1.80–3.59)	<0.001
Waist circumference, per SD	1.99	(1.67–2.38)	<0.001	2.21	(1.86–2.69)	<0.001	2.21	(1.84–2.66)	<0.001
Waist criterion^a^, yes	4.12	(2.15–7.92)	<0.001	4.14	(2.14–7.99)	<0.001	4.22	(2.18–8.16)	<0.001
Triglycerides, per SD	1.40	(1.19–1.64)	<0.001	1.43	(1.21–1.69)	<0.001	1.41	(1.19–1.67)	<0.001
Triglyceride criterion^a^, yes	2.01	(1.37–2.93)	<0.001	2.1	(1.42–3.09)	<0.001	2.06	(1.40–3.03)	<0.001
HDL, per SD lower	1.55	(1.26–1.84)	<0.001	1.70	(1.38–2.09)	<0.001	1.68	(1.36–2.07)	<0.001
HDL criterion^a^, yes	1.70	(1.14–2.55)	0.01	1.74	(1.16–2.62)	<0.01	1.69	(1.12–2.55)	0,012
Glucose, per SD	1.46	(1.24–1.71)	<0.001	1.51	(1.28–1.80)	<0.001	1.52	(1.29–1.80)	<0.001
Glucose citerion^a^, yes	1.74	(1.24–2.45)	0.001	1.82	(1.28–2.61)	0.001	1.84	(1.29–2.63)	0.001
Systolic blood pressure, per SD	1.09	(0.92–1.29)	0.32	1.09	(0.92–1.30)	0.30	1.11	(0.94–1.33)	0.22
Blood pressure criterion^a^, yes	1.11	(0.79–1.55)	0.56	1.10	(0.77–1.56)	0.61	1.13	(0.79–1.60)	0.51

HDL, high density lipoprotein; MetS, metabolic syndrome; OR, odds ratio.

All variables were analyzed in separate regression models.

Model 1: crude.

Model 2 adjusted for age, sex and height.

Model 3: model 2 + current smoking.

a Dichotomized variables: waist circumference ≥ 94 cm in men and ≥ 80 for women, glucose ≥ 5.6 mmol/l, SBT ≥ 130 mm Hg, DBT ≥ 85mm Hg or use of antihypertensive medication, or a combination of these, triglycerides ≥ 1.7 mmol/l or use of TG-altering drugs, HDL <1.03 in men or <1.29 in women or use of HDL-altering drugs.

We repeated the analyses using the National Institutes of Health MetS definition (waist circumference threshold ≥102 cm in men and ≥88 cm in women) and the consensus MetS definition in which waist circumference was not mandatory (Supplementary Tables S5–S8).^35^ The results were almost the same. There was no significant sex interaction for the association between MetS and absolute GFR or hyperfiltration.

The association of MetS, fasting glucose, and waist circumference with GFR was attenuated using the BSA-corrected GFR (ml/min per 1.73 m^2^) (Supplementary Table S9). The association with hyperfiltration using a definition based on age-specific and sex-specific cut-offs for BSA-adjusted GFR (in ml/min per 1.73 m^2^) was no longer significant for MetS but remained significant for fasting glucose and the triglyceride criterion, in the fully adjusted model (Supplementary Table S10).

### Associations Between MetS and Hyperfiltration Using eGFRcrea, eGFRcys, and eGFRcreacys

MetS was not associated with higher baseline eGFR or hyperfiltration based on eGFRcrea, eGFRcys, or eGFRcreacys when hyperfiltration was defined using age-specific and sex-specific cut-offs (eGFR normalized by BSA) (Supplementary Table S11). Conversely, for eGFRcys, higher triglycerides, lower HDL-cholesterol, and the blood pressure criterion were associated with a lower OR of hyperfiltration (Supplementary Table S11). However, when hyperfiltration was defined using age-specific, sex-specific, and height-specific cut-offs for absolute eGFR (in ml/min), we observed similar associations between hyperfiltration and metabolic risk factors as with the mGFR (Supplementary Table S12).

### The Associations Between MetS, hyperfiltration, and Long-Term GFR Decline

The mean annual mGFR change rates among all participants was −0.95 ml/min per 1.73 m^2^ (95% CI: −0.87 to −1.04) in women and −1.21 ml/min per 1.73 m^2^ (−1.12 to −1.30) in men. MetS was associated with a steeper GFR decline rate in crude and adjusted linear mixed models (models 1 and 2, Table 4). There was no significant sex interaction. The association was attenuated but still significant after additional adjustment for baseline hyperfiltration (model 3, Table 4). Compared to those without MetS, GFR decline was steeper in those with MetS and hyperfiltration than in those with MetS and no hyperfiltration (−0.83 [95% CI: −1.13 to −0.53] ml/min per 1.73 m^2^ per year vs. −0.15 [−0.30 to 0.00]) (Table 4). However, there was no significant interactions between MetS and hyperfiltration on GFR change rates (*P* = 0.2 and *P =* 0.4 in models 1 and 2, respectively).

**Table 4:** Associations between metabolic syndrome and GFR decline rates during 11 years of follow-up in linear mixed model

No MetS vs. MetS	Model 1			Model 2			Model 3		
	beta	95% CI	*P* value	beta	95% CI	*P* value	beta	95% CI	*P* value
No MetS	ref	(−0.43 to −0.16)		ref	(−0.40 to −0.12)		ref		
MetS	−0.30		<0.001	−0.26		<0.001^a^	−0.18		0.01
No MetS	ref	(−0.34 to −0.04)		ref	(−0.30 to 0.00)			(−0.32 to −0.04)	
MetS without hyperfiltration	−0.19		0.01	−0.15		0.05			
MetS with hyperfiltration	−0.84	(−0.15 to −0.54)	<0.001^b^	−0.83	(−1.13 to −0.53)	<0.001^b^			

MetS, metabolic syndrome.

Model 1: crude (unadjusted).

Model 2: adjusted for age, sex, height, and current smoking, including their interaction with time (effect on the GFR decline rate).

Model 3: model 2 + hyperfiltration at baseline, including the interaction between hyperfiltration and time.

a *P*-value= 0.4 for interaction of sex and MetS on GFR decline.

b *P*-value = 0.2 and 0.4 for interactions of MetS and hyperfiltration on GFR decline rates in model 1 and 2.

## Discussion

In middle-aged subjects from the general nondiabetic population, MetS was associated with higher mGFR and hyperfiltration. The GFR and OR of hyperfiltration at baseline increased with the increasing number of metabolic risk factors, particularly when the waist circumference, glucose, and triglyceride criteria were met. MetS was strongly associated with a steeper GFR decline in those with hyperfiltration and moderately associated with a steeper GFR decline in those without hyperfiltration.

Previous studies on MetS and hyperfiltration have reported inconsistent results and were all based on eGFR to assess kidney function.^10^, ^11^, ^12^, ^13^ In a study of 1572 healthy young men, in which the kidney function was assessed by creatinine clearance (ml/min) using the Cockroft-Gault formula, they found that MetS and the components blood pressure, body mass index, and low HDL levels, but not glucose and triglycerides, were associated with hyperfiltration. Although indexing the GFR for BSA obscures a genuine association between obesity and GFR, the Cockroft-Gault formula is known to overestimate the GFR in obese subjects.^11^^,^^20^

Although the best method to determine hyperfiltration is unknown, it has been suggested that the definition of hyperfiltration should be adjusted for age and sex to account for nephron number.^30^ In a study of the adult Japanese population, hyperfiltration was defined as eGFRcrea (ml/min per 1.73 m^2^) above the age-specific and sex-specific 95th percentile. MetS was associated with a 17% increased risk of hyperfiltration (OR 1.2, [1.1–1.2]) and among the MetS components, only increased blood pressure and fasting glucose were associated with hyperfiltration, whereas higher triglycerides and waist circumference, and lower HDL, were associated with lower odds of hyperfiltration.^12^

More recently, Chakkera *et al.*^31^ investigated methodological aspects related to studies of hyperfiltration by including biopsy-verified markers of single nephron hyperfiltration. They found that the 95th percentile thresholds for eGFR were considerably lower than that for mGFR (118 ml/min per 1.73 m^2^ vs. 134 ml/min per 1.73 m^2^), and high mGFR was a much better marker of single nephron hyperfiltration than eGFR. A high age-height-gender-based absolute GFR definition of hyperfiltration was the best-suited method to differentiate between biopsy-verified markers of single nephron hyperfiltration and high total GFR in persons with higher nephron number.^31^ The nephron number decreases with age and is lower in females and in people with lower stature.^32^ A noncorrected threshold for hyperfiltration would therefore mask associations with hyperfiltration on a glomerular level, particularly in women, older adults, and in those with lower stature.^39^ Accordingly, we adjusted our definition of renal hyperfiltration for age, sex, and height and included these covariates in the regression models with absolute GFR and hyperfiltration.

Approximately one-third of middle-aged persons have MetS. In a previous RENIS study, we found that MetS was associated with a steeper GFR decline during a median of 5.6 years of follow-up.^26^ A recent meta-analysis of cohort studies found that persons with MetS, on average, have a 34% increased risk of incident CKD (OR 1.34 [1.28–1.39]). However, the risk of CKD was attenuated and borderline significant in most studies when persons with diabetes were excluded.^40^ In the current study, MetS was associated with baseline hyperfiltration and subsequent long-term GFR decline using accurate GFR measurements. Furthermore, we found that the subgroup of persons with MetS and hyperfiltration had a much steeper GFR decline rate.

Our findings have several clinical implications. Treatment with sodium-glucose cotransporter-2 inhibitors prevented hyperfiltration and was renoprotective in patients with diabetes and/or CKD.^21^^,^^22^ Therefore, randomized controlled trials using sodium-glucose cotransporter-2 inhibitors in persons with MetS and hyperfiltration are warranted. Nondrug treatment such as a low-protein diet and physical exercise may also reduce hyperfiltration and should be considered for all persons with MetS.^41^, ^42^, ^43^

Our study illustrates that it is difficult to identify persons with hyperfiltration for risk assessment and inclusion in trials. Hyperfiltration is not easily detected by eGFR because it is commonly indexed by BSA, lacks precision in the high-normal range, and is biased by non-GFR related factors.^16^, ^17^, ^18^, ^19^, ^20^

We found that participants with MetS had a higher mean mGFR, also when indexed for BSA, but a lower mean eGFRcrea, eGFRcys and eGFRcreacys, compared to the non-MetS group (Table 2). However, when hyperfiltration was defined according to absolute eGFR (recalculated CKD-EPI equation to absolute GFR in ml/min) and then adjusted for age, sex, and height, we obtained similar associations with hyperfiltration as in the mGFR analyses. Therefore, in studies of hyperfiltration using eGFR, absolute eGFR adjusted for age, sex, and height, maybe a better proxy for single nephron hyperfiltration caused by metabolic risk factors than the commonly used BSA-indexed eGFR.

Increased body weight is an integral part of MetS, and it is therefore difficult to disentangle the effect of body weight and MetS on the risk of hyperfiltration. In 2 previous studies in RENIS, we investigated the association of fasting glucose and abdominal obesity and hyperfiltration using a definition that was corrected for age, sex, height, and body weight. Stefansson *et al.*^8^ found that waist-hip ratio was a better obesity measure than body mass index and waist circumference to uncover hyperfiltration independently of body weight. Although the whole-kidney absolute GFR increases when individuals gain weight, the nephron number does not increase; single nephron GFR also increases accordingly. Therefore, in this study of MetS, we chose not to adjust for body weight in our primary analyses because it would mask the association between MetS and hyperfiltration at a glomerular level.^19^^,^^20^

The main strength of this study is the use of repeated measurements of GFR instead of eGFR in a cohort representative of the general middle-aged nondiabetic population. In addition, we used a definition of hyperfiltration that may be more closely correlated to single-nephron hyperfiltration than the definitions used by most previous studies.

The study also has limitations. The GFR decline rate in participants with MetS and hyperfiltration at baseline may be influenced by the regression to the mean in those with high baseline GFR. However, we used a linear mixed model that accounts for baseline GFR levels and random effects, supporting a true association of hyperfiltration with accelerated GFR decline in MetS. We did not measure the effective renal plasma flow and could not investigate the role of filtration fraction in hyperfiltration. Our study population was composed of middle-aged Caucasians, which limits the generalizability to other ethnicities or age groups.

We conclude that MetS is associated with hyperfiltration and accelerated GFR decline in the general nondiabetic population. Among the MetS risk factors, increased waist circumference, impaired fasting glucose, high triglycerides, and low HDL-cholesterol were associated with hyperfiltration. Randomized trials targeting hyperfiltration are warranted to assess whether accelerated GFR decline, CKD, and premature death can be prevented in subjects with MetS.

## Disclosure

All the authors declared no competing interests.

## Supplementary Material

Supplementary File (PDF)

**Table S1.** Consensus definition of Mets.

**Table S2.** Association of GFR (ml/min) with MetS and its components.

**Table S3.** Odds ratio for hyperfiltration by number of MetS risk factors.

**Table S4.** Odds ratio for hyperfiltration by number of the following MetS risk factors; the waist criterion, glucose criterion and triglyceride criterion.

**Table S5.** Association of GFR (ml/min) with MetS and its components with NIH cut-off for waist circumference.

**Table S6.** Associations of hyperfiltration with MetS and its components (absolute GFR) with NIH cut-off for waist circumference.

**Table S7.** Association of GFR (ml/min) with the MetS-using the consensus definition of MetS∗.

**Table S8.** Associations of hyperfiltration with MetS (absolute GFR)-using the consensus definition of MetS∗.

**Table S9.** Associations of MetS and its components with GFR indexed by BSA.

**Table S10.** Association of MetS and its components with hyperfiltration (BSA adjusted).

**Table S11.** Association of hyperfiltration with MetS and its components (age-and sex-specific eGFR cut-off indexed by BSA).

**Table S12.** Associations of hyperfiltration with MetS and its components (age-, sex-, and height-specific cut-off of absolute eGFR).

**STROBE Statement.**
