# Corrigendum to “The Association Between Metabolic Syndrome, Hyperfiltration, and Long-Term GFR Decline in the General Population” [*Kidney International Reports* Volume 8, Issue 9, September 2023, Pages 1831-1840]

**DOI:** 10.1016/j.ekir.2024.05.002

**Published:** 2024-05-06

**Authors:** Erikka W. Bystad, Vidar T.N. Stefansson, Bjørn O. Eriksen, Toralf Melsom

**Affiliations:** 1 Metabolic and Renal Research Group, UiT-The Arctic University of Norway, Tromsø, Norway; 2 Department of Neurology, University Hospital of North Norway, Tromsø, Norway; 3 Section of Nephrology, Clinic of Internal Medicine, University Hospital of North Norway, Tromsø, Norway

The authors regret that there was a small error in the calculation of iohexol clearance (mGFR) in the third round of the Renal Iohexol Clearance Survey (RENIS-3).

The initially reported mean (SD) iohexol clearance in RENIS-3 utilized was 82.0 (15.1) mL/min/1.73 m^2^, but the revised mean (SD) should be 82.0 (14.4) mL/min/1.73 m^2^.

The authors would like to apologize for any inconvenience caused.

DOI of original article: https://doi.org/10.1016/j.ekir.2023.06.022

