# Supplementary material for: The Association Between Metabolic Syndrome, Hyperfiltration, and Long-Term GFR Decline in the General Population
Source: Kidney Int Rep. 2023 Jul 1;8(9):1831–40. doi: 10.1016/j.ekir.2023.06.022 (PMC10496074; doi:10.1016/j.ekir.2023.06.022)
Supplement: Supplementary File (PDF) [file mmc1.pdf]

Supplementary Material

Table of contents

STROBE Statement 2

Supplemental Table S1: Consensus definition of MetS*.. 4

Supplemental Table S2: Association of GFR (mL/min) with MetS and its components.. 5

Supplemental Table S3: Odds ratio for hyperfiltration by number of MetS risk factors 6

Supplemental Table S4: Odds ratio for hyperfiltration by number of the following MetS risk factors; the waist criterion, glucose criterion, and triglyceride criterion 7

Supplemental Table S7: Association of GFR (mL/min) with the MetS - using the consensus definition of MetS* 9

Supplemental Table S8: Associations of hyperfiltration with MetS (absolute GFR) - using the consensus definition of MetS* 9

Supplemental Table S9: Associations of MetS and its components with GFR indexed by BSA 10

Supplemental Table S10: Association of MetS and its components with hyperfiltration (BSA adjusted) 11

Supplemental Table S11: Association of hyperfiltration with MetS and its components (age-and sex-specific eGFR cut-off indexed by BSA) 12

Supplemental Table S12: Associations of hyperfiltration with MetS and its components (age-, sex-, and height-specific cut-off of absolute eGFR) 13

STROBE Statement—Checklist of items that should be included in reports of *cohort studies*

	Item No	Recommendation	Page No
**Title and abstract**	1	(*a*) Indicate the study's design with a commonly used term in the title or the abstract	2
		(*b*) Provide in the abstract an informative and balanced summary of what was done and what was found	2
**Introduction**			
Background/rationale	2	Explain the scientific background and rationale for the investigation being reported	3-4
Objectives	3	State specific objectives, including any prespecified hypotheses	4
**Methods**			
Study design	4	Present key elements of study design early in the paper	2,4,5
Setting	5	Describe the setting, locations, and relevant dates, including periods of recruitment, exposure, follow-up, and data collection	4, 5
Participants	6	(*a*) Give the eligibility criteria, and the sources and methods of selection of participants. Describe methods of follow-up	4, 5
		(*b*) For matched studies, give matching criteria and number of exposed and unexposed	
Variables	7	Clearly define all outcomes, exposures, predictors, potential confounders, and effect modifiers. Give diagnostic criteria, if applicable	5-8
Data sources/ measurement	8*	For each variable of interest, give sources of data and details of methods of assessment (measurement). Describe comparability of assessment methods if there is more than one group	5-7
Bias	9	Describe any efforts to address potential sources of bias	6, 7
Study size	10	Explain how the study size was arrived at	4, 5
Quantitative variables	11	Explain how quantitative variables were handled in the analyses. If applicable, describe which groupings were chosen and why	5-7
Statistical methods	12	(*a*) Describe all statistical methods, including those used to control for confounding	7, 8
		(*b*) Describe any methods used to examine subgroups and interactions	7, 8
		(*c*) Explain how missing data were addressed	4 (Table 2)
		(*d*) If applicable, explain how loss to follow-up was addressed	
		(*e*) Describe any sensitivity analyses	7,8
**Results**			

Participants	13*	(a) Report numbers of individuals at each stage of study—eg numbers potentially eligible, examined for eligibility, confirmed eligible, included in the study, completing follow-up, and analysed	4, 5, 8, 9, Figure 1
		(b) Give reasons for non-participation at each stage	4, 5,<br>Figure 1
		(c) Consider use of a flow diagram	Figure 1
Descriptive data	14*	(a) Give characteristics of study participants (eg demographic, clinical, social) and information on exposures and potential confounders	8, 9,<br>Table 2
		(b) Indicate number of participants with missing data for each variable of interest	Table 2 (no missing)
		(c) Summarise follow-up time (eg, average and total amount)	9
Outcome data	15*	Report numbers of outcome events or summary measures over time	8, 9, Table 2
Main results	16	(*a*) Give unadjusted estimates and, if applicable, confounder-adjusted estimates and their precision (eg, 95% confidence interval). Make clear which confounders were adjusted for and why they were included	8-11
		(*b*) Report category boundaries when continuous variables were categorized	8-11
		(*c*) If relevant, consider translating estimates of relative risk into absolute risk for a meaningful time period	9, 10
Other analyses	17	Report other analyses done—eg analyses of subgroups and interactions, and sensitivity analyses	
**Discussion**			
Key results	18	Summarise key results with reference to study objectives	11
Limitations	19	Discuss limitations of the study, taking into account sources of potential bias or imprecision. Discuss both direction and magnitude of any potential bias	15
Interpretation	20	Give a cautious overall interpretation of results considering objectives, limitations, multiplicity of analyses, results from similar studies, and other relevant evidence	11-15
Generalisability	21	Discuss the generalisability (external validity) of the study results	14-15
**Other information**			
Funding	22	Give the source of funding and the role of the funders for the present study and, if applicable, for the original study on which the present article is based	16

Supplemental Table S1: Consensus definition of MetS*

- Elevated waist circumference: ≥ 94 cm in men and ≥ 80 cm in women[a]
- Fasting plasma glucose ≥ 5.6 mmol/L
- Systolic blood pressure ≥ 130 mmHg, diastolic blood pressure ≥ 85 mmHg or use of antihypertensive medication, or a combination of these
- Triglycerides ≥ 1.7 mmol/L or use of triglyceride-altering drugs
- HDL-cholesterol < 1.0 mmol/L in men or < 1.3 in women or use of HDL-altering drugs

MetS metabolic syndrome, *HDL* high-density lipoprotein

*A consensus definition incorporating the International Diabetes Federation (IDF) and the American Heart Association/National Heart, Lung, and Blood Institute (AHA/NHLBI) definition, where fulfilling three out of five criteria qualifies for the metabolic syndrome.(1)

[a]Population- and country-specific definitions should be used according to the consensus definition

Supplemental Table S2: Association of GFR (mL/min) with MetS and its components

	Model 1			Model 2			Model 3		
	Coef.	CI	P	Coef.	CI	P	Coef.	CI	P
Metabolic syndrome, yes	10.73	(8.66-12.79)	<0.001	6.73	(5.02-8.45)	<0.001	6.68	(4.97-8.39)	<0.001
Waist circumference, per SD	9.02	(8.15-9.90)	<0.001	5.69	(4.86-6.51)	<0.001	5.71	(4.89-6.54)	<0.001
Waist-criterion[a]	5.05	(2.63-7.48)	<0.001	8.39	(6.45-10.33)	<0.001	8.49	(6.54-10.43)	<0.001
Triglycerides, per SD	3.73	(2.76-4.70)	<0.001	1.82	(1.02-2.62)	<0.001	1.71	(0.91-2.52)	<0.001
Triglyceride criterion[a]	9.12	(6.55-11.69)	<0.001	4.54	(2.44-6.64)	<0.001	4.39	(2.29-6.50)	<0.001
HDL, per SD lower	6.01	(5.07-6.94)	<0.001	2.29	(1.45-3.12)	<0.001	2.21	(1.38-3.05)	<0.001
HDL criterion[a]	6.17	(3.46-8.88)	<0.001	3.94	(1.76-6.13)	<0.001	3.73	(1.54-5.92)	0.001
Fasting glucose, per SD	7.30	(6.22-8.37)	<0.001	4.01	(3.07-4.94)	<0.001	4.03	(3.09-4.97)	<0.001
Glucose criterion[a]	10.23	(8.12-12.34)	<0.001	5.19	(3.41-6.97)	<0.001	5.20	(3.42-6.98)	<0.001
Systolic blood pressure, per SD	2.76	(1.78-3.74)	<0.001	0.80	(-0.04-1.64)	0.061	0.89	(0.05-1.73)	0.038
Blood pressure criterion[a]	3.61	(1.61-5.60)	<0.001	0.44	(-1.22-2.10)	0.604	0.59	(-1.08-2.26)	0.491

GFR glomerular filtration rate, *MetS* metabolic syndrome, HDL high density lipoprotein, *SD* standard deviation, *Coef* coefficient, *CI* confidence interval

All variables were analysed in separate regression models

[a]Dichotomized variables: waist circumference ≥ 94 cm in men and ≥ 80 for women, glucose ≥ 5.6 mmol/L, SBT ≥ 130 mmHg, DBT ≥ 85mmHg or use of antihypertensive medication, or a combination of these, triglycerides ≥ 1.7 mmol/L or use of TG-altering drugs, HDL < 1.03 in men or <1.29 in women or use of HDL-altering drugs.

Model 1: crude

Model 2 adjusted for age, sex and height

Model 3: model 2 + current smoking

Supplemental Table S3: Odds ratio for hyperfiltration by number of MetS risk factors

	Model 1			Model 2			Model 3		
Number of MetS risk factors	OR	CI	P	OR	CI	P	OR	CI	P
0-1[a]	ref			ref			ref		
2	1.41	(0.89-2.28)	0.143	1.45	(0.09-2.34)	0.125	1.48	(0.92-2.38)	0.108
3	2.86	(1.80-4.56)	<0.001	3.05	(1.89-4.93)	<0.001	3.09	(1.92-5.00)	<0.001
4	2.35	(1.25-4.44)	0.008	2.56	(1.34-4.88)	0.004	2.51	(1.31-4.80)	0.005
5	2.63	(1.39-9.44)	0.008	4.06	(1.54-10.67)	0.005	4.16	(1.58-10.99)	0.004
Linear trend	1.42	(1.23-1.64)	<0.001	1.46	(1.26-1.70)	<0.001	1.46	(1.25-1.69)	<0.001

MetS metabolic syndrome, *OR* odds ratio, *CI* confidence interval

[a]Because the number of participants with hyperfiltration in the group without any MS risk factor (group 0) was less than 5 (4 people), we merged groups 0 and 1 in the logistic regression analyses.

Model 1: crude

Model 2: age, sex, height

Model 3: age, sex, height, current smoking

Supplemental Table S4: Odds ratio for hyperfiltration by number of the following MetS risk factors; the waist criterion, glucose criterion and triglyceride criterion

		Model 1			Model 2			Model 3		
Number of MetS risk factors	People	OR	CI	P	OR	CI	P	OR	CI	P
0	N=240	ref			ref			ref		
1	N=779	2.83	(1.27-6.27)	0.01	2.73	(1.23-6.07)	0,01	2.75	(1.24-6.11)	0.01
2	N=428	5.75	(2.59-12.76)	<0.001	5.96	(2.67-13.29)	<0.001	6.04	(2.71-13.48)	<0.001
3	N=104	6.97	(2.81-17.26)	<0.001	7.30	(2.93-18.18)	<0.001	7.23	(2.90-18.03)	<0.001

MetS metabolic syndrome, *OR* odds ratio, *CI* confidence interval
Model 1: crude
Model 2: age, sex, height
Model 3: age, sex, height, current smoking

Supplemental Table S5: Association of GFR (mL/min) with MetS and its components with NIH cut-off for waist circumference

	Model 1			Model 2			Model 3		
	Coef.	CI	P	Coef.	CI	P	Coef.	CI	P
Metabolic syndrome	10.01	(7.79-12.23)	<0.001	7.43	(5.63-9.24)	<0.001	7.23	(5.52-9.13)	<0.001
Waist criterion[a]	3.41	(1.44-5.37)	0.001	8.02	(6.44-9.61)	<0.001	8.05	(6.47-9.63)	<0.001

GFR glomerular filtration rate, *MetS* metabolic syndrome, *NIH* National Institutes of Health, *Coef.* coefficient, *CI* confidence interval
All variables were analysed in separate regression models
[a]Dichotomized variables: waist circumference ≥102 cm in men and ≥ 88 for women
Model 1: crude
Model 2 adjusted for age, sex and height
Model 3: model 2 + current smoking

Supplemental Table S6: Associations of hyperfiltration with MetS and its components (absolute GFR) with NIH cut-off for waist circumference

	Model 1			Model 2			Model 3		
	OR	CI	P	OR	CI	P	OR	CI	P
Metabolic syndrome	2.63	(1.88-3.71)	<0.001	2.69	(1.90-3.80)	<0.001	2.65	(1.87-3.75)	<0.001
Waist criterion[a]	3.37	(2.31-4.92)	<0.001	3.52	(2.38-5.19)	<0.001	3.52	(2.39-5.20)	<0.001

GFR glomerular filtration rate, *MetS* metabolic syndrome, *NIH* National Institutes of Health, *OR* odds ratio, *CI* confidence interval
All variables were analysed in separate regression models
[a]Dichotomized variables: waist circumference ≥ 102 cm in men and ≥ 88 for women
Model 1: crude
Model 2 adjusted for age, sex and height
Model 3: model 2 + current smoking

Supplemental Table S7: Association of GFR (mL/min) with the MetS - using the consensus definition of MetS*

	Model 1			Model 2			Model 3		
	Coef.	CI	P	Coef.	CI	P	Coef.	CI	P
Metabolic syndrome, yes	10.57	(8.51-12.64)	<0.001	6.37	(4.65-8.08)	<0.001	6.31	(4.61-8.02)	<0.001

GFR glomerular filtration rate, *MetS* metabolic syndrome, *Coef.* coefficient, *CI* confidence interval
All variables were analysed in separate regression models
*A consensus definition incorporating the International Diabetes Federation and the American Heart Association/National Heart, Lung, and Blood Institute definition, where fulfilling three out of five criteria qualifies for the metabolic syndrome.(1)
Model 1: crude
Model 2 adjusted for age, sex and height
Model 3: model 2 + current smoking

Supplemental Table S8: Associations of hyperfiltration with MetS (absolute GFR) - using the consensus definition of MetS*

	Model 1			Model 2			Model 3		
	OR	CI	P	OR	CI	P	OR	CI	P
Metabolic syndrome, yes	2.38	(1.70-3.33)	<0.001	2.5	(1.77-3.53)	<0.001	2.49	(1.76-3.52)	<0.001

GFR glomerular filtration rate, *MetS* metabolic syndrome, *OR* odds ratio, *CI* confidence interval
All variables were analysed in separate regression models
*A consensus definition incorporating the International Diabetes Federation and the American Heart Association/National Heart, Lung, and Blood Institute definition, where fulfilling three out of five criteria qualifies for the metabolic syndrome.(1)
Model 1: crude
Model 2 adjusted for age, sex and height
Model 3: model 2 + current smoking

Supplemental Table S9: Associations of MetS and its components with GFR indexed by BSA

	Crude			Adjusted[b]		
	Coef.	CI	P	Coef.	CI	P
Metabolic syndrome	2.09	(0.55 to 3.63)	<0.01	1.82	(0.22 to 3.42)	<0.05
Waist circumference	0.95	(0.23 to 1.66)	<0.01	1.41	(0.01 to 2.80)	<0.05
Waist criterion[a]	-2.15	(-3.92 to -0.39)	0.02	0.96	(-0.99 to 2.90)	0.34
Triglycerides	0.35	(-0.37 to 1.06)	0.35	-0.47	(-1.2 to 0.27)	0.21
Triglyceride criterion[a]	1.95	(0.06 to 3.84)	0.04	0.59	(-1.25 to 2.43)	0.53
HDL	1.49	(0.78 to 2.20)	<0.001	-0.04	(-0.81 to 0.72)	0.91
HDL criterion[a]	1.36	(-0.62 to 3.34)	0.18	0.57	(-1.33 to 2.46)	0.56
Glucose	2.40	(1.58 to 3.21)	<0.001	1.87	(1.02 to 2.71)	<0.001
Glucose criterion[a]	3.41	(1.84 to 5.00)	<0.001	2.35	(0.81 to 3.90)	<0.01
Systolic blood pressure	-0.09	(-0.80 to 0.63)	0.82	-0.49	(-1.22 to 0.24)	0.18
Blood pressure criterion[a]	-1.47	(-2.93 to -0.02)	<0.05	-2.15	(-3.59 to -0.71)	<0.01

MetS metabolic syndrome, *GFR* glomerular filtration rate, *BSA* body surface area *HDL* high density lipoprotein, *Coef* coefficient, *CI* confidence interval

All variables were analysed in separate regression models

[a]Dichotomized variables: waist circumference ≥ 94 cm in men and ≥ 80 for women, glucose ≥ 5.6 mmol/L, SBT ≥ 130 mmHg, DBT ≥ 85mmHg or use of antihypertensive medication, or a combination of these, triglycerides ≥ 1.7 mmol/L or use of TG-altering drugs, HDL < 1.03 in men or <1.29 in women or use of HDL-altering drugs

[b]Adjusted for age, sex, height, current smoking (yes/no) and body weight.

Supplemental Table S10: Association of MetS and its components with hyperfiltration (BSA adjusted)

	**Crude**			**Adjusted[b]**		
	Coef.	CI	P	Coef.	CI	P
Metabolic syndrome	1.26	(0.89-1.79)	0.19	1.39	(0.94-2.07)	0.10
Waist circumference	1.03	(0.87-1.22)	0.72	1.43	(1.00-2.03)	0.05
Waist criterion[a]	0.99	(0.66-1.50)	0.98	1.28	(0.78-2.10)	0.32
Triglycerides	1.13	(0.96-1.33)	0.14	1.1	(0.91-1.32)	0.30
Triglyceride criterion[a]	1.46	(0.98-2.19)	0.06	1.54	(1.00-2.38)	0.05
HDL	1.18	(0.99-1.40)	0.07	1.19	(0.98-1.46)	0.09
HDL criterion[a]	1.43	(0.94-0.2.17)	0.10	1.42	(0.91-2.22)	0.12
Glucose	1.26	(1.08-1.49)	0.004	1.35	(1.12-1.61)	0.001
Glucose criterion[a]	1.27	(0.90-1.81)	0.18	1.31	(0.90-1.92)	0.16
Systolic blood pressure	1.01	(0.85-1.19)	0.93	1.01	(0.84-1.21)	0.91
Blood pressure criterion[a]	0.85	(0.66-1.29)	0.33	0.83	(0.57-1.19)	0.31

MetS metabolic syndrome, *BSA* body surface area, *HDL* high-density lipoprotein

Hyperfiltration was defined as subjects above the age-and sex-specific 90th percentile of GFR (indexed by BSA).

[a]Dichotomized variables: waist circumference ≥ 94 cm in men and ≥ 80 for women, glucose ≥ 5.6 mmol/L, SBT ≥ 130 mmHg, DBT ≥ 85mmHg or use of antihypertensive medication, or a combination of these, triglycerides ≥ 1.7 mmol/L or use of TG-altering drugs, HDL < 1.03 in men or <1.29 in women or use of HDL-altering drugs

[b]Logistic regression model was adjusted for age, sex, height, current smoking (yes/no), and body weight.

Supplemental Table S11: Association of hyperfiltration with MetS and its components (age-and sex-specific eGFR cut-off indexed by BSA)

	eGFRcrea			eGFRcys			eGFRcreacys		
	OR	CI	P	OR	CI	P	OR	CI	P
Metabolic syndrome, yes	0.97	(0.65-1.47)	0.9	0.71	(0.45-1.11)	0.14	0.91	(0.59-1.40)	0.68
Waist circumference, per SD	1.44	(1.01-2.06)	0.04	1.07	(0.75-1.54)	0.68	1.35	(0.95-1.93)	0.09
Waist-criterion[a]	1.04	(0.64-1.69)	0.86	1.26	(0.78-2.01)	0.34	1.30	(0.80-2.10)	0.29
Triglycerides, per SD	0.93	(0.77-1.12)	0.44	0.75	(0.61-0.91)	0.004	0.80	(0.66-0.98)	0.27
Triglyceride criterion[a]	0.79	(0.48-1.30)	0.36	0.43	(0.23-0.82)	0.01	0.50	(0.28-0.92)	0.03
HDL, per SD lower	1.04	(0.86-1.27)	0.67	0.82	(0.68-0.99)	0.036	0.91	(0.75-1.09)	0.29
HDL criterion[a]	1.17	(0.74-1.86)	0.032	0.76	(0.43-1.33)	0.34	0.79	(0.46-1.35)	0.40
Glucose, per SD	1.02	(0.85-1.22)	0.82	1.17	(0.97-1.41)	0.1	1.21	(1.01-1.46)	0.04
Glucose criterion[a]	0.94	(0.63-1.40)	0.77	1.22	(0.82-1.79)	0.33	1.29	(0.87-1.90)	0.21
Systolic blood pressure, per SD	1.03	(0.86-1.23)	0.78	0.86	(0.71-1.04)	0.12	0.93	(0.77-1.11)	0.41
Blood pressure criterion[a]	0.87	(0.60-1.24)	0.45	0.68	(0.47-0.97)	0.03	0.77	(0.54-1.11)	0.16

MetS metabolic syndrome, *eGFR* estimated glomerular filtration rate, *eGFRcrea* eGFR based on creatinine, *eGFRcys* eGFR based on cystatin, *eGFRcreacys* eGFR based on creatinine and cystatin, *BSA* body surface area, *HDL* high-density lipoprotein, *SD* standard deviation
Adjusted for age, sex, height, body weight, and current smoking.
[a]Dichotomized variables: waist circumference ≥ 94 cm in men and ≥ 80 for women, glucose ≥ 5.6 mmol/L, SBT ≥ 130 mmHg, DBT ≥ 85mmHg or use of antihypertensive medication, or a combination of these, triglycerides ≥ 1.7 mmol/L or use of TG-altering drugs, HDL < 1.03 in men or <1.29 in women or use of HDL-altering drugs

Supplemental Table S12: Associations of hyperfiltration with MetS and its components (age-, sex-, and height-specific cut-off of absolute eGFR)

	eGFRcrea			eGFRcys			eGFRcreacys		
	OR	CI	P	OR	CI	P	OR	CI	P
Metabolic syndrome, yes	2.35	(1.68-3.31)	<0.001	2.49	(1.75-3.53)	<0.001	2.48	(1.74-3.52)	<0.001
Waist circumference, per SD	1.99	(1.67-2.38)	<0.001	2.24	(1.86-2.69)	<0.001	2.24	(1.86-2.69)	<0.001
Waist-criterion[a]	3.93	(2.04-7.56)	<0.001	3.93	(2.04-7.60)	<0.001	4.00	(2.07-7.75)	<0.001
Triglycerides, per SD	1.40	(1.19-1.64)	<0.001	1.43	(1.21-1.69)	<0.001	1.41	(1.19-1.67)	<0.001
Triglyceride criterion[a]	2.05	(1.40-3.02)	<0.001	2.15	(1.45-3.19)	<0.001	2.12	(1.43-3.13)	<0.001
HDL, per SD lower	1.52	(1.26-1.84)	<0.001	1.67	(1.36-2.06)	<0.001	1.65	(1.34-2.03)	<0.001
HDL criterion[a]	1.58	(1.04-2.40)	0.032	1.61	(1.06-2.46)	0.025	1.57	(1.03-2.39)	0.038
Glucose, per SD	1.57	(1.30-1.90)	<0.001	1.67	(1.37-2.03)	<0.001	1.67	(1.38-2.04)	<0.001
Glucose criterion[a]	1.76	(1.24-2.49)	0.001	1.87	(1.30-2.69)	0.001	1.89	(1.31-2.71)	0.001
Systolic blood pressure, per SD	1.09	(0.92-1.29)	0.320	1.1	(0.92-1.31)	0.297	1.12	(0.94-1.33)	0.224
Blood pressure criterion[a]	1.06	(0.75-1.50)	0.738	1.06	(0.74-1.52)	0.738	1.09	(0.76-1.56)	0.637

MetS metabolic syndrome, *eGFR* estimated glomerular filtration rate, *eGFRcrea* eGFR based on creatinine, *eGFRcys* eGFR based on cystatin, *eGFRcreacys* eGFR based on creatinine and cystatin, *HDL* high-density lipoprotein, *SD* standard deviation

Adjusted for age, sex, height, body weight, and current smoking.

[a]Dichotomized variables: waist circumference ≥ 94 cm in men and ≥ 80 for women, glucose ≥ 5.6 mmol/L, SBT ≥ 130 mmHg, DBT ≥ 85mmHg or use of antihypertensive medication, or a combination of these, triglycerides ≥ 1.7 mmol/L or use of TG-altering drugs, HDL < 1.03 in men or <1.29 in women or use of HDL-altering drugs

1. Alberti KGMM, Eckel RH, Grundy SM, Zimmet PZ, Cleeman JI, Donato KA, et al. Harmonizing the Metabolic Syndrome. Circulation. 2009;120(16):1640-5.
